# Entirely thoracoscopic resection of a giant emphysematous bulla

**DOI:** 10.11604/pamj.2018.30.247.12400

**Published:** 2018-08-06

**Authors:** Agustín Buero, Walter Sebastian Nardi, Gustavo Alejandro Lyons, Silvia Quadrelli, Domingo Javier Chimondeguy

**Affiliations:** 1 British Hospital of Buenos Aires, Buenos Aires, Argentina

**Keywords:** giant bulla, bullectomy, VATS

## Abstract

A 38-year-old man with longilinear shape, smoker (38 packs/year) and no other relevant medical history was referred to our department due to the finding of left pulmonary hyperlucency on a chest x-ray. A computed tomography (CT) was performed and a giant emphysematous bulla with thin-walled partitions inside was shown that replaced almost the entire left upper lobe, The patient underwent an exploratory thoracoscopy. Intraoperatively a giant bulla was seen that initially impressed to replace the entire upper lobe. Despite the large size we decided to attempt thoracoscopic resection preserving the remaining healthy parenchyma. Bullectomy was done using linear endoscopic stapling devices. To our knowledge this is the only case with such a large bulla resected entirely by VATS.

## Introduction

Giant bullous emphysema (GBE) was first described by Burke in 1937 [1] and involves the presence of emphysematous areas with complete destruction of lung tissue producing airspaces bigger than 1cm in diameter. The bulls area does not participates in broncho-alveolar oxygenation and can cause symptoms such as dyspnea, thoracic pain, infection, pneumothorax and even slow progression to malignancy. Roberts et al described radiographic criteria for this entity: giant bullae in one or both upper lobes occupying at least one third of the hemithorax and compressing the normal surrounding parenchyma [2]. Surgery is indicated in symptomatic patients and in asymptomatic patients who have a bulla that occupies more than one-third of the hemithorax or when in the clinical follow-up an increase in the size of the bulla is observed. We report herein the case of a patient with a giant bulla who underwent successful bullectomy.

## Patient and observation

**Figure 1:**
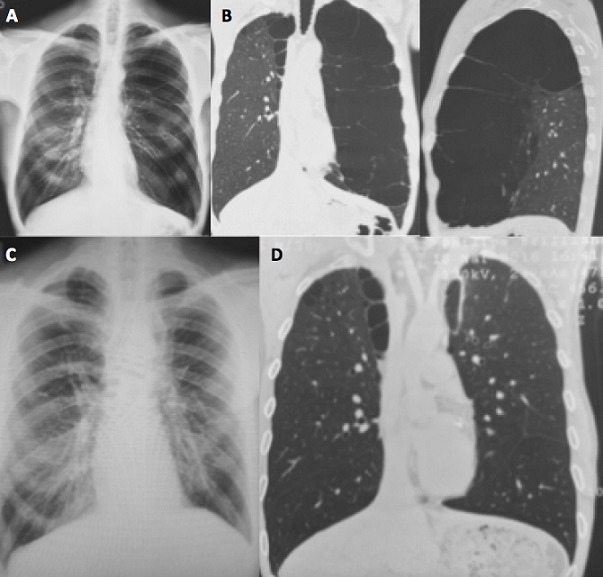
A) preoperative chest X-Ray. Left pulmonary hyperlucency; B) preoperative chest CT. Giant emphysematous bulla with thin-walled partitions inside replacing almost the entire upper lobe; C) postoperative chest X-Ray. Complete pulmonary expansion; D) postoperative chest CT

**Figure 2:**
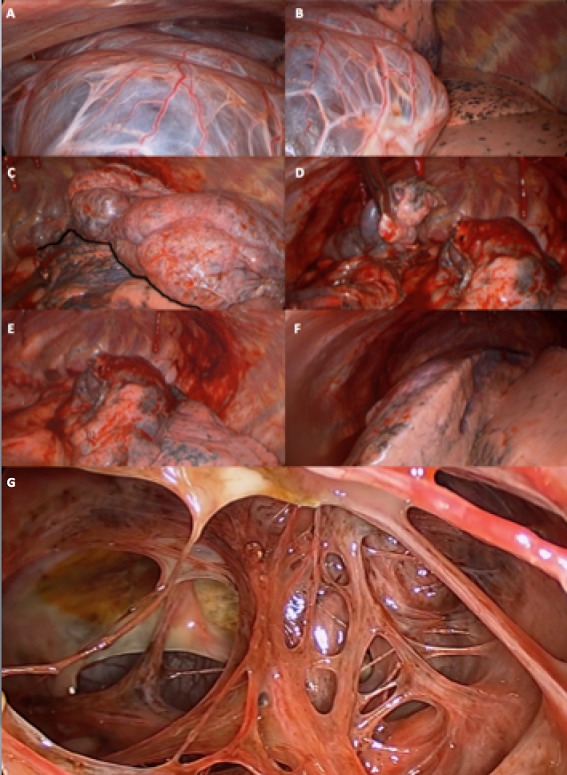
A) giant bull occupying; B) upper lobe almost replaced by the giant bulla. Healthy lower lobe; C) upper lobe was partially affected persisting a healthy parenchyma area. Black line: implantation line of the bulla; (D, E) bullectomy performed with endoscopic stapling devices; F) lung expansion of the remnant; G) interior of the bulla

A 38-year-old man with longilinear shape, smoker (38 packs/year) with no other relevant medical history was referred to our department due to the finding of left pulmonary hyperlucency on a chest x-ray done (Figure 1A) because an upper airway disease. A computed tomography (CT) was performed and a giant emphysematous bulla with thin-walled partitions inside was shown, that replaced almost the entire left upper lobe (Figure 1B). Preoperative spirometry revealed a forced vital capacity (FVC) of 5.5lts (99%) and forced expiratory volume on the 1st second (FEV1) of 3.27lts (71%). Carbon monoxide diffusion was 6.84 (58%). The dosage of alpha-1 antitrypsin was within the reference values. The patient underwent an exploratory thoracoscopy to evaluate the extension of the bulla. Intraoperatively we saw a giant bulla that initially impressed to replace the entire upper lobe. Once all firm adhesions to parietal pleura were released and the bulla was opened for a better dissection, because of its large size, we saw that the upper lobe was partially affected persisting a healthy parenchyma area. Despite the large size we decided to attempt thoracoscopic resection trying to preserve the remaining healthy parenchyma. Bullectomy was done using six linear endoscopic stapling devices of 60mm. Pulmonary insufflation after resection showed a good lung expansion of the remnant (Figure 2 A-G). Finally mechanical pleural abrasion was done. Pleural cavity was drained with two chest tubes connected to a water seal. Histopathology examination revealed a giant cavitated emphysematous bulla with walls 0.1cm thick. Patient presented lack of lung expansion on chest x-ray with persistent air loss trough both drains. Continuous aspiration was placed. He remained with pleural drainage for 13 days (Figure 1C). A postoperative spirometry and CT showed an improvement of all the volumes and carbon monoxide diffusion (FVC 6.07lts -108%, FEV1 5.31lts -116%, DLCO 9.42 - 80%) and complete pulmonary expansion (Figure 1D).

## Discussion

GBE, sometimes referred to as Vanishing Lung Syndrome (VLS) as a clinical syndrome, was first described by Burke in 1937. It typically presents in young males cigarette smokers as in our patient and also can be associated with other conditions (alpha-1 antitrypsin deficiency or marijuana abuse). Development of multiple large lung bullae (termed GBE or VLS) is a rare and progressive disease with chest radiographs showing peripheral (paraseptal) bullae in contrast to the more uniformly distributed bullae of centrilobular emphysema shown in smokers. High resolution computed tomography (HRCT) typically shows extensive paraseptal emphysema coalescing into giant bullae and is the best imaging to determine the extension of the disease. Surgery is indicated to treat complications related to GBE or in asymptomatic patients who have a bulla that occupies more than one-third of the hemithorax compressing healthy adjacent lung tissue and when in the clinical follow-up an increase in the size of the bulla is observed. Different thoracoscopic techniques are reported in literature and almost all are nowadays abandoned [3-5]. The VATS bullectomy with endoscopic staple resection is considered a safe and feasible procedure when possible. We consider that total resection must be done since the higher risk in patients with GBE to develop bronchogenic carcinoma [6, 7]. The most common post-operative complication following bullectomy is persistent air leakage, which can prolong hospital stay [8, 9]. In spite of this, it did no prevent the compressed lung tissue from re-expanding. Some authors recommend the use of pericardial strips to prevent the air leakage in the case of lung volume reduction for severe emphysema [10]. Other authors suggest associating a pleurectomy in order to achieve a better pleurodesis and reduce air leaking.

## Conclusion

We presented the only case with a bulla of such size resected completely by videothoracoscopy. To obtain satisfactory results, both postoperative and operative care are essential. As we mentioned, the most frequent complication is persistent air leakage and the lack of pulmonary expansion in the postoperative period. The use of adequate mechanical sutures, some type of pleurodesis and mechanical and respiratory kinesiology are key points in this type of interventions. Despite there is not enough evidence published with similar cases resolved entirely by VATS, we consider that it's a safe and feasible procedure in experienced centers.

## Competing interests

The authors declare no competing interests.
